# Olfactory Dysfunction Predicts Disease Progression in Parkinson’s Disease: A Longitudinal Study

**DOI:** 10.3389/fnins.2020.569777

**Published:** 2020-12-14

**Authors:** Runcheng He, Yuwen Zhao, Yan He, Yangjie Zhou, Jinxia Yang, Xiaoting Zhou, Liping Zhu, Xun Zhou, Zhenhua Liu, Qian Xu, Qiying Sun, Jieqiong Tan, Xinxiang Yan, Beisha Tang, Jifeng Guo

**Affiliations:** ^1^Department of Neurology, Xiangya Hospital, Central South University, Changsha, China; ^2^Department of Geriatrics, Xiangya Hospital, Central South University, Changsha, China; ^3^Key Laboratory of Hunan Province in Neurodegenerative Disorders, Central South University, Changsha, China; ^4^National Clinical Research Center for Geriatric Disorders, Xiangya Hospital, Central South University, Changsha, China; ^5^Center for Medical Genetics, School of Life Sciences, Central South University, Changsha, China

**Keywords:** Parkinson’s disease, olfactory dysfunction, hyposmia, disease progression, cognitive impairment

## Abstract

**Background and Objective:**

Olfactory dysfunction (hyposmia) is an important non-motor symptom of Parkinson’s disease (PD). To investigate the potential prognostic value of hyposmia as a marker for disease progression, we prospectively assessed clinical manifestations and longitudinal changes of hyposmic PD patients and normosmic ones.

**Methods:**

Olfactory function was evaluated with the Sniffin’ Sticks in PD patients at baseline. One hundred five hyposmic PD patients and 59 normosmic PD patients were enrolled and followed up for 2 years. They were subsequently evaluated at baseline and during follow-up periods with neurological and neuropsychological assessments. Clinical manifestations and disease progressions were compared between hyposmic and normosmic patients. In addition, the relationship between disease progressions and olfactory function was analyzed.

**Results:**

Our study suggested that hyposmic PD patients and normosmic ones were similar in gender, age, education levels, age of onset, disease duration, and clinical features at baseline. Hyposmic PD patients exhibited more severe Unified Parkinson’s Disease Rating Scale Part II–III (UPDRS II-III) scores, higher levodopa equivalent dose (LED) needs, and poorer Mini-Mental State Examination (MMSE) score at follow-up visits compared to those in normosmic PD patients. Hyposmia also showed greater rates in the increase of LED needs, improvement of UPDRS III score, and deterioration of MMSE score. Both improvement of UPDRS III score and decline of MMSE score were associated with poorer odor identification.

**Conclusion:**

Our prospective study demonstrated that hyposmic PD patients showed a relatively worse clinical course compared with normosmic patients. Olfactory dysfunction is a useful predictor of disease progression.

## Introduction

Parkinson’s disease (PD) is one of the common progressive neurodegenerative disorders characterized by several motor and non-motor symptoms (NMSs). Olfactory dysfunction is the most common NMS in PD patients and often predates motor symptoms (Ansari and Johnson, 1975; He et al., 2018; Masala et al., 2018b; Haehner et al., 2019). Olfactory dysfunction, as one of the supportive criteria for PD, has been incorporated in the MDS Clinical Diagnostic Criteria for Parkinson’s Disease (Postuma et al., 2015).

The mechanism of olfactory dysfunction in PD remains currently unclear, but it is believed to be related to the α-synuclein aggregates in peripheral and central olfactory structures. The synucleinopathy density scores in the olfactory bulb are correlated with scores on the Unified Parkinson’s Disease Rating Scale Part III (UPDRS III) (Beach et al., 2009a). Rey et al. (2016) triggered α-synuclein pathology in the olfactory bulb of mice by local injections of fibrillar α-synuclein, and they found that it can gradually spread to a total of over 40 different brain regions or subregions that are also affected in PD over the course of 12 months. Eventually, they detected α-synuclein inclusions in neocortical brain regions (Rey et al., 2016). As a result, olfactory dysfunction may be a clinical marker of disease progression. Cross-sectional studies reported that olfactory dysfunction is connected with motor symptoms severity. PD patients with high olfactory function are milder and may progress more slowly (Berendse et al., 2011; Masala et al., 2018a). Olfactory dysfunction is also correlated with cognitive impairment in PD, and it can increase the risk of dementia up to 10 years after PD diagnosis (Domellof et al., 2017; Cecchini et al., 2019).

Olfactory function could play a significant role in the evaluation of disease progression and cognitive decline. Several studies have investigated olfaction in PD patients. However, most studies are limited in assessing disease progression differences because they are cross-sectional or retrospective. We report the longitudinal follow-up assessment of PD patients in early and middle stages, with or without olfactory dysfunction, followed up prospectively for up to 2 years. The aim of our study was to investigate the relationship between olfactory function and disease progression.

## Materials and Methods

### Participants

The PD patients were recruited from the inpatients and outpatients of the Department of Neurology of Xiangya Hospital, Central South University, between January 2016 and December 2017 at Parkinson’s Disease and Movement Disorders Multicenter Database and Collaborative Network in China (PD-MDCNC)^1^, and ethical approval was obtained from the Medical Ethics Committee of Xiangya Hospital. Each patient was diagnosed with clinically established PD or clinically probable PD by at least two experienced neurologists according to Movement Disorder Society (MDS) diagnostic criteria (Postuma et al., 2015). Only patients performing the Sniffin’ Sticks test were included in the current study. Because the advanced PD patients were not the target cohort of the present research project, PD patients at Hoehn–Yahr (H-Y) stages IV and V were excluded. All participants provided written informed consent prior to participating in this study.

**FIGURE 1:**
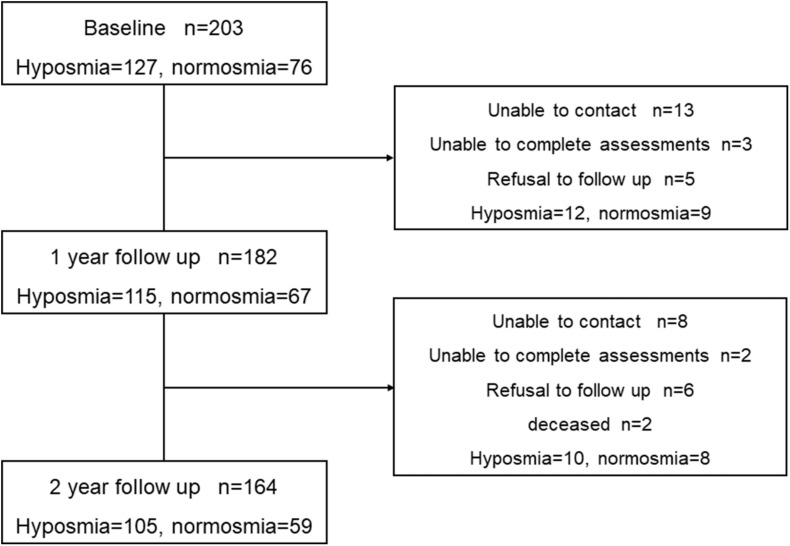
Flow chart of patients with Parkinson’s disease included in the study.

At baseline, a total of 203 consecutive participants without dementia were identified as having available data. According to the standard of Hummel et al. (2007), 127 (62.6%) participants were classified as hyposmic PD patients, and 76 (37.4%) participants were classified as normosmic PD patients. We conduct a prospective comprehensive assessment of these participants every year from January 2017 to December 2019. There were 115 hyposmic PD patients and 67 normosmic ones at year 1, and 2-year data were available for 105 (64.0%) hyposmia (mean age 56.91, SD 10.04) and 59 (36.0%) normosmia (mean age 59.49, SD 9.18). Over a 2-year follow-up, 39 patients did not return for follow-up visits. Finally, 164 PD patients who were evaluated with the same assessments at baseline and at 2-year follow-up were enrolled in our prospective study (Figure 1).

### Assessments

Clinical data including gender, age, education level, age at onset (AAO), disease duration, and levodopa equivalent dose (LED) were collected by interview. All participants subsequently were evaluated at baseline and yearly with neurological and neuropsychological assessments.

Motor symptoms were evaluated by the UPDRS PartII, UPDRS Part III, and H-Y Scale. All participants were evaluated in the OFF state at baseline and in the ON state at follow-up visits. Tremor score was calculated by adding up scores of tremor at rest (UPDRS III-20) and action and postural tremor of hands (UPDRS III-21). Bradykinesia score was calculated by added up scores on finger taps (UPDRS III-23), hand movements (UPDRS III-24), rapid alternating movements of hand (UPDRS III-25), and leg agility (UPDRS III-26). Rigidity score was measured by the score on UPDRS III-22. Postural and gait abnormalities score was measured by the sum of UPDRS III-27, UPDRS III-28, UPDRS III-29, and UPDRS III-30 score. According to the ratio of the mean tremor score (including UPDRS II-16, UPDRS III-20, and UPDRS III-21) to the mean postural instability and gait difficulty (PIGD) score (including UPDRS II-13, UPDRS II-14, UPDRS II-15, UPDRS III-29, and UPDRS III-30), we classified the enrolled PD patients into tremor-dominant (TD) phenotype, PIGD phenotype, and indeterminate phenotype. Patients with a ratio of no more than 1.0 were defined as PIGD, while those with a ratio of no less than 1.5 were categorized as TD, and the rest of the patients were classified as indeterminate phenotype (Xiang et al., 2019). Freezing was evaluated by UPDRS II-14. Wearing-off was evaluated by the Wearing-Off Questionnaire-9 (WOQ-9), and dyskinesia was evaluated by the Rush Dyskinesia Rating Scale and UPDRS IV (Zhou et al., 2019).

Non-motor symptoms scale (NMSS) was used to assess the severity of NMSs. NMSS contains nine dimensions: cardiovascular, sleep/fatigue, mood/cognition, perceptual problems, attention/memory, gastrointestinal, urinary, sexual function, and miscellany. Cognitive function was evaluated by the Mini-Mental State Examination (MMSE), with scores ranging from 0 to 30 (Wang et al., 2015). The cutoff points for dementia screening were 17 for illiterate, 20 for individuals with 1–6 years of education, and 24 for individuals with 7 or more years of education (Li et al., 2016). Rapid eye movement sleep behavior disorder (RBD) was assessed by the REM Sleep Behavior Disorder Questionnaire–Hong Kong (RBDQ-HK), and the cutoff value for factor 2 of RBDQ-HK was 7, and that for the RBDQ-HK overall scale was 17 (Shen et al., 2014). The Hamilton Rating Scale for Depression (HAMD-17) was applied to assess depression.

We assessed olfactory performance using the Sniffin’ Sticks test. Sniffin’ Sticks were applied to assess the odor threshold, odor discrimination, and odor identification. Odors were presented in felt-tip pens, and the pens were consecutively placed approximately 2 cm in front of both nostrils with a 20-s interval between odor presentations. Odor threshold test (T score) was applied to evaluate the ability to perceive the lowest concentration of an odor. Odor discrimination test (D score) was applied to assess the ability to differentiate two different odors. Odor identification test (I score) was applied to measure the ability to choose the correct odor from a list of four descriptors (Hummel et al., 1997). The “TDI score” equals to the sum of results obtained for threshold, discrimination, and identification tests. Since the olfactory function decreases in relation to age as indicated in previous studies (Doty and Kamath, 2014; Masala et al., 2018a), the cutoff value for the TDI score was 30.3 for subjects aged 36–55 years and 19.6 for subjects older than 55 years. Subjects were classified as normosmic if they reached the above score, and hyposmic if less than the cutoff value (Hummel et al., 2007; Zhao et al., 2020).

### Statistical Analysis

For the cross-sectional analyses at baseline and follow-up, means ± standard deviations were used to describe continuous variables, while percentages and frequencies were used for describing categorical variables. Prior to statistical comparisons, we performed Shapiro–Wilk Normality Test on the given data. Since these data do not conform to normal distribution, the continuous variables of hyposmic PD patients and normosmic ones at baseline were compared using the non-parametric Mann–Whitney *U* tests, and the binary variables were compared using the chi-square tests. In the longitudinal analyses, Wilcoxon matched-pairs signed-rank test was used to assess the within-group differences of continuous variables, and McNemar tests or McNemar–Bowker tests were used for categorical variables. Relationships between the changes of UPDRS III/MMSE score over time and Sniffin’ Sticks scores were assayed with partial correlation analysis, and age and UPDRS III/MMSE score at baseline were adjusted. The linear mixed-effect models were used to compare rates of disease progression between two groups over time. Moreover, univariate and multivariate linear regression analyses were performed to identify predictors of UPDRS III and MMSE scores at follow-up visit. For the multivariate linear regression analysis, UPDRS III score at follow-up visit was considered as dependent variables, while independent variables were gender, age, disease duration, olfactory function, H-Y stage, and disease severity by UPDRS III at baseline. And MMSE score at follow-up visit was considered as a dependent variable, while independent variables were gender, age, disease duration, education levels, H-Y stage, UPDRS III score, and MMSE score at baseline. Significance was considered if *P* < 0.05. Statistical analysis was performed using SPSS version 22.0.

## Results

In our study, 105 (64.0%) participants were classified as hyposmic PD patients, and 59 (36.0%) participants were classified as normosmic PD patients. The demographic and clinical characteristics of hyposmic and normosmic PD patients are presented in Table 1. Specifically, there were no significant differences between hyposmic patients and normosmic ones in terms of gender, age, education levels, AAO, or disease duration. The average TDI score for the hyposmic and normosmic PD patients were 16.70 ± 6.22 and 25.92 ± 4.39 points. Mean T score, D score, and I score of hyposmia were significantly lower than normosmia (*P* < 0.001). At baseline, there were no significant differences between two groups in UPDRS I–III subscale scores, proportion of motor subtypes, H-Y scale, LED needs, NMSS total scores, NMSS subscores, proportion of dyskinesia, or freezing.

**TABLE 1:** Comparison of demographic and clinical characteristics between the hyposmic PD patients and normosmic PD patients at baseline.

Items	Hyposmic (*n* = 127)	Normosmic (*n* = 76)	*P* value
Gender (male%)	66 (52.0%)	34 (45.0%)	0.220
Age (years)	56.91 ± 10.04	59.49 ± 9.18	0.524
Education years (years)	9.50 ± 4.04	9.87 ± 3.87	0.527
AAO (years)	53.09 ± 10.04	55.66 ± 8.92	0.067
Duration (years)	4.57 ± 3.85	3.88 ± 2.91	0.172
TDI score	16.48 ± 6.56	25.53 ± 4.90	**<0.001**
T score	4.12 ± 2.97	7.28 ± 3.38	**<0.001**
D score	6.59 ± 2.93	9.13 ± 2.46	**<0.001**
I score	5.80 ± 2.59	9.12 ± 2.16	**<0.001**

*Data for continuous variables are presented as mean ± standard deviation. Values in bold refer to statistically significant differences (*P* < 0.05). AAO, age at onset; PD, Parkinson’s disease; TDI, threshold, discrimination, and identification.*

### Longitudinal Changes in Motor Characteristics in Hyposmia and Normosmia

Levodopa equivalent dose needs increased from baseline 334.86 to 560.21 mg at year 2 in hyposmic PD patients (*P* < 0.001) vs. from 275.00 to 465.15 mg in normosmic patients (*P* < 0.001), and mean LED needs in hyposmic patients were significantly higher than normosmic ones at year 2 (*P* = 0.020) (Table 2). The increase rate of LED needs in hyposmic patients was greater compared to that in normosmic ones (*P* < 0.001). Both hyposmic and normosmic PD patients exhibited improvement from baseline in UPDRS Part II and III subscale scores during the 2-year follow-up period (*P* < 0.05) (Table 2). There was a significant difference in the rate of improvement of UPDRS III scores between normosmia (better) and hyposmia within the first year (*P* = 0.010). Compared with normosmic patients, hyposmic patients had worse UPDRS III score, rigidity score, bradykinesia score, and H-Y stage at year 1 (Table 2). Normosmic patients showed significant improvements from baseline in rigidity, bradykinesia, and postural and gait abnormalities scores at year 1 (*P* = 0.046, *P* = 0.016, and *P* = 0.042) with a return to baseline scores at year 2 (*P* = 0.605, *P* = 0.283, and *P* = 0.411). The improvement in UPDRS III score at first year was correlated with the TDI score (*r* = 0.161, *P* = 0.041) and I score (*r* = 0.174, *P* = 0.026), after adjusting for age and UPDRS III score at baseline (Table 3). Univariate and multivariate regression analyses revealed that gender (male), disease duration, olfactory function, and UPDRS III score at baseline were the significant predictors of UPDRS III score at year 1 (Table 4). Male sex, olfactory dysfunction, longer disease duration, and more severe disease at baseline were significantly associated with higher UPDRS III score at year 2. Adjusted R^2^ of multivariate regression analysis was 0.337 for it. Neither hyposmic nor normosmic PD patients demonstrated a significant longitudinal increase in H-Y stage over time. Hyposmic patients showed significant declines from baseline in bradykinesia and postural and gait abnormalities scores at year 2 (*P* = 0.011, *P* = 0.022). For hyposmic group, the prevalence of PIGD at the second year (65.7%) was noticeably higher than that at baseline (54.3%) (*P* = 0.027). Although there was a trend toward a higher proportion of PIGD in the hyposmic group compared with that in the normosmic one, there were no significant differences in the proportions of motor subtypes between the two groups.

**TABLE 2:** PD motor and non-motor characteristics of the hyposmic PD patients and normosmic PD patients over time.

	Baseline			Year 1			Year 2		
			
Variable	Hyposmic	Normosmic	*P* value	Hyposmic	Normosmic	*P* value	Hyposmic	Normosmic	*P* value
UPDRS subscores								^*b*^	
Part I	2.36 ± 1.90	2.69 ± 2.13	0.347	2.14 ± 1.86	2.02 ± 1.59	0.907	2.56 ± 1.85	2.36 ± 1.83	0.410
Part II	12.05 ± 5.96	11.97 ± 5.58	0.917	10.47 ± 5.73^*a*^	9.47 ± 4.62^*a*^	0.304	10.59 ± 4.80^*b*^	10.69 ± 6.31^*b*^	0.761
Part III	25.70 ± 12.65	26.17 ± 14.13	0.999	23.26 ± 11.88^*a*^	19.00 ± 9.70^*a*^	**0.030**	22.98 ± 12.67^*b*^	22.37 ± 13.67^*b*^	0.672
Tremor	3.32 ± 3.04	2.78 ± 2.67	0.367	2.85 ± 3.00	2.83 ± 2.89	0.999	2.86 ± 2.66	2.95 ± 3.54	0.444
Rigidity	5.74 ± 3.85	4.64 ± 3.67	0.051	5.18 ± 3.64	3.37 ± 2.75^*a*^	**0.002**	4.73 ± 3.92^*b*^	4.56 ± 4.12	0.717
Bradykinesia	8.82 ± 6.01	8.85 ± 6.58	0.831	8.47 ± 5.15	6.62 ± 4.03^*a*^	**0.013**	8.87 ± 5.98	7.56 ± 6.05	0.125
Postural and gait abnormalities	3.66 ± 2.16	3.66 ± 2.88	0.552	3.10 ± 2.24^*a*^	2.75 ± 1.89^*a*^	0.327	3.11 ± 2.59^*b*^	3.32 ± 2.64	0.612
Phenotype (PIGD/Intermediate/TD)	58/12/35	33/8/18	0.888	67/10/28	32/6/21	0.450	71/13/21^*b*^	40/6/13	0.889
H-Y Stage	2.00 ± 0.65	2.02 ± 0.72	0.915	2.14 ± 0.64 ^*a*^	1.90 ± 0.75	**0.027**	2.13 ± 0.70^*b*^	2.05 ± 0.87	0.365
Dyskinesia (%)	16 (15.2%)	4 (6.8%)	0.112	23 (21.9%)	9 (15.3%)	0.302	30 (28.6%)^*b*^	8 (13.6%)	**0.029**
Freezing (%)	37 (35.2%)	16 (27.1%)	0.286	31 (29.5%)	11 (18.6%)	0.126	35 (33.3%)	12 (20.3%)	0.077
LED (mg)	334.86 ± 259.54	275.00 ± 194.60	0.166	469.22 ± 249.90^*a*^	394.19 ± 212.68^*a*^	0.104	560.21 ± 253.55^*b*^	465.15 ± 197.49**^*b*^**	**0.020**
NMSS total scores	52.92 ± 38.04	46.76 ± 31.49	0.490	35.43 ± 24.81^*a*^	28.88 ± 19.96^*a*^	0.132	33.59 ± 27.54^*b*^	31.20 ± 21.29^*b*^	0.949
Cardiovascular	1.54 ± 2.77	1.46 ± 2.61	0.870	0.78 ± 1.78^*a*^	0.73 ± 1.80^*a*^	0.899	0.62 ± 1.54^*b*^	1.00 ± 2.09	0.149
Sleep/fatigue	11.57 ± 9.66	10.24 ± 8.89	0.396	8.18 ± 7.21	7.68 ± 6.45	0.796	8.33 ± 7.73	9.86 ± 7.18	0.451
Mood/cognition	10.68 ± 10.80	9.98 ± 11.19	0.612	5.85 ± 8.31^*a*^	3.85 ± 6.03^*a*^	0.178	4.81 ± 8.67^*b*^	5.24 ± 9.81^*b*^	0.619
Perceptual problems	1.11 ± 2.41	0.73 ± 2.04	0.357	0.95 ± 2.04	0.80 ± 1.74	0.558	0.89 ± 2.12	1.15 ± 1.94	0.127
Gastrointestinal	4.10 ± 5.31	4.47 ± 5.54	0.489	3.61 ± 4.34	3.34 ± 4.26	0.609	4.28 ± 5.17	3.25 ± 4.03	0.354
Urinary	7.70 ± 8.16	6.73 ± 7.58	0.557	5.60 ± 6.16^*a*^	4.47 ± 5.36^*a*^	0.224	5.17 ± 6.08^*b*^	5.51 ± 6.57	0.939
MMSE total scores	27.31 ± 2.49	27.80 ± 1.76	0.430	27.15 ± 3.03	27.66 ± 2.19	0.537	26.99 ± 2.89	27.90 ± 2.35	**0.041**
Orientation	9.55 ± 0.91	9.66 ± 0.55	0.919	9.70 ± 0.77	9.80 ± 0.48	0.743	9.66 ± 0.83	9.71 ± 0.62	0.935
Registration	2.79 ± 0.41	2.81 ± 0.51	0.387	2.85 ± 0.48	2.92 ± 0.28	0.620	2.89 ± 0.32	2.93 ± 0.25	0.337
Attention and calculation	4.22 ± 1.04	4.34 ± 0.96	0.096	3.81 ± 1.39^*a*^	4.00 ± 1.33	0.357	3.73 ± 1.42^*b*^	4.17 ± 1.21	**0.048**
Recall	2.37 ± 0.86	2.34 ± 0.96	0.520	2.45 ± 0.83	2.36 ± 0.80	0.310	2.38 ± 0.76	2.49 ± 0.70	0.378
Language and praxis	8.32 ± 0.96	8.58 ± 0.59	0.982	8.35 ± 1.05	8.59 ± 0.72	0.216	8.33 ± 0.92	8.59 ± 0.77	0.054
RBD (%)	39 (37.1%)	15 (25.4%)	0.125	43 (41.0%)	22 (37.3%)	0.645	51 (48.6%)	25 (42.4%)	0.445
Depression (%)	30 (28.6%)	17 (28.8%)	0.974	26 (24.8%)	13 (19.4%)	0.674	29 (27.6%)	21 (35.6%)	0.287

*Values in bold refer to statistically significant differences (*P* < 0.05). ^*a*^Significant difference between baseline and year 1 (*P* < 0.05). ^*b*^Significant difference between baseline and year 2 (*P* < 0.05). UPDRS, Unified Parkinson’s Disease Rating Scale; H-Y, Hoehn and Yahr; PIGD, postural instability and gait disorder; TD, tremor dominant; LED, levodopa equivalent dose; NMSS, non-motor symptoms scale; MMSE, Mini-Mental State Examination; RBD, rapid eye movement sleep behavior disorder; PD, Parkinson’s disease.*

**TABLE 3:** Correlation coefficient between the improvement in UPDRS III score from baseline to year 1 and Sniffin’ Sticks score.

Variable	Correlation coefficient	*P* value
TDI score	0.161	**0.041**
T score	0.104	0.190
D score	0.092	0.246
I score	0.174	**0.026**

*Adjusted for age and UPDRS III at baseline. Values in bold refer to statistically significant differences (*P* < 0.05). TDI, threshold, discrimination, and identification; UPDRS, Unified Parkinson’s Disease Rating Scale.*

**TABLE 4:** Multivariate linear regression analysis of clinical factors associated with UPDRS III score at year 1.

Predictors	Beta	*P* value
Gender (male)	0.130	**0.044**
Age	0.092	0.160
Disease duration	0.230	**0.001**
Olfactory function (olfactory dysfunction)	0.152	**0.020**
H-Y Stage	0.058	0.484
UPDRS III Score at baseline	0.432	**<0.001**

*Values in bold refer to statistically significant differences (*P* < 0.05). H-Y, Hoehn and Yahr; UPDRS, Unified Parkinson’s Disease Rating Scale.*

The proportion of hyposmic PD patients with dyskinesia increased over time (*P* = 0.001), and the prevalence of dyskinesia in hyposmic patients was higher than that in normosmic group at year 2 (*P* = 0.029) (Table 2). But there was no difference in the proportion of PD patients with dyskinesia among the two groups (*P* = 0.227) after adjusting for disease duration and LED needs. Meanwhile, there were no significant longitudinal changes in frequency of freezing in the two groups (*P* > 0.012).

### Longitudinal Changes in Non-motor Characteristics in Hyposmia and Normosmia

There were no significant differences in UPDRS Part I (neuropsychiatric), NMSS, or NMSS subscores between the two groups at any time point (Table 2). Both hyposmic and normosmic PD patients demonstrated a significant longitudinal increase in the severity of NMSS over time. There were no significant differences in the rate of improvement of NMSS score between the two groups within 1 (*P* = 0.167) or 2 years (*P* = 0.839). When the various domains were analyzed, significant improvements were present in the following domains: Cardiovascular, Mood/cognition, and Urinary. There was no significant difference in the frequency of depression between the two groups at any time point. And it showed similar rates of change over time.

Although the prevalence of RBD in hyposmic patients was relatively high, there was no significant difference between groups for the prevalence of RBD at any time point (Table 2). To determine baseline clinical variables that predict incident development of RBD, we evaluated the 110 PD patients in this cohort who did not have RBD at baseline. Cox proportional hazards analysis showed that UPDRS III score at baseline (controlled for age, AOO, and gender) increased the risk of RBD 1.021 times [hazard ratio (HR) (95% CI) = 1.021 (1.001–1.042), *P* = 0.003]. Hyposmia did not increase the risk of RBD (*P* = 0.363).

There were no significant between-group differences in MMSE or MMSE subscores at baseline (Table 2). At 2 follow-up time points, hyposmic patients had worse MMSE total score (*P* = 0.041, Cohen’s d = 0.170) as well as attention and calculation subscore (*P* = 0.048, Cohen’s d = 0.164), with no significant difference in orientation, registration, recall, and language and praxis scores. In the longitudinal analysis, the hyposmic patients had significant longitudinal deterioration in total MMSE scores (*P* < 0.001), particularly in attention and calculation scores (*P* < 0.001), from baseline to year 2, whereas normosmia demonstrated no significant longitudinal worsening. The rate of deterioration in hyposmic patients was greater than that in normosmic patients (controlled for age) (*P* = 0.029). The longitudinal decline in MMSE score from baseline to year 2 was correlated with the TDI score (*r* = −0.164, *P* = 0.037) and I score (*r* = 0.170, *P* = 0.030), after adjusting for age and MMSE score at baseline (Table 5). Univariate and multivariate regression analyses revealed that age, olfactory function, UPDRS III score, and MMSE score at baseline were the significant predictors of MMSE score at year 2 (Table 6). Older age, olfactory dysfunction, more severe disease, and worse cognitive function at baseline were significantly associated with lower MMSE score at year 2. Adjusted R^2^ of the multivariate regression analysis was 0.422 for it.

**TABLE 5:** Correlation coefficient between the decrease in MMSE score from baseline to year 2 and Sniffin’ Sticks score.

Variable	Correlation coefficient	*P* value
TDI score	−0.164	**0.037**
T score	−0.076	0.339
D score	−0.139	0.078
I score	−0.170	**0.030**

*Adjusted for age and MMSE score at baseline. Values in bold refer to statistically significant differences (*P* < 0.05). MMSE, Mini-Mental State Examination; TDI, threshold, discrimination, and identification.*

**TABLE 6:** Multivariable linear regression analysis of clinical factors associated with MMSE score at year 2.

Predictors	Beta	*P* value
Gender (male)	0.083	0.179
Age	−0.062	**0.001**
Disease duration	−0.072	0.267
Education levels	−0.019	0.755
Olfactory function (olfactory dysfunction)	−0.792	**0.023**
H-Y stage	−0.053	0.484
UPDRS III score at baseline	−0.026	**0.043**
MMSE score at baseline	0.578	**<0.001**

*Values in bold refer to statistically significant differences (*P* < 0.05). H-Y, Hoehn and Yahr; MMSE, Mini-Mental State Examination; UPDRS, Unified Parkinson’s Disease Rating Scale.*

## Discussion

The purpose of our study was to observe the longitudinal evolution of motor and non-motor characteristics in the cohort of hyposmic and normosmic PD patients. According to Braak staging, anterior olfactory nucleus and olfactory bulb are first affected by Lewy body pathology. With the involvement of additional nuclear grays, the pathology moves up the brainstem, and it reaches the substantia nigra by Braak stage 3 (Braak et al., 2003). Therefore, fewer pathologies in the olfactory bulb might be associated with less pathologic spreading into the brain, and hyposmia may be useful as a marker of disease progression.

Previous study suggested that the α-synucleinopathy density in the olfactory bulb are associated with UPDRS III scores (Beach et al., 2009b). Our study showed that hyposmic PD patients had significantly higher disease severity (as measured by UPDRS III and H-Y stage) and LED needs than those in normosmic PD patients. The effects of dopaminergic medications on hyposmic and normosmic PD patients were different. Compared with hyposmic PD patients, motor symptoms (as measured by UPDRS III) in normosmic PD patients were more levodopa-responsive at first. Greater improvement in motor symptoms was associated with better odor identification and general olfactory function. Normosmic patients had greater improvement in rigidity and bradykinesia under medication. The improvement in motor symptoms was associated with the olfactory function, especially the odor identification performance. Over time, motor symptoms progressively became levodopa-resistant. Our result is in line with previous study that found that normosmic PD patients had better motor function and a more benign course than hyposmic ones (Lee et al., 2015). More benign disease process of normosmia may be associated with less pathologic spread into the brain from the olfactory bulb. However, this is in contrast with a previous study that suggested that olfactory dysfunction was not associated with motor function or LED needs (Rossi et al., 2016). This discrepancy may be caused by the fact that the patients in their study were at the earlier stage of the disease and their severity of the disease was milder. As we all know, the quality of life is severely impaired by olfactory dysfunction. Kang et al. (2012) found that olfactory scores of the University of Pennsylvania Smell Identification Test (UPSIT) were negatively associated with Parkinson’s Disease Questionnaire 39 (PDQ-39) and positively correlated with Schwab and England activities of daily living score. Zhao et al. (2020) indicated that worse daily living ability was related to lower olfactory identification scores. The pathophysiological basis of olfactory deficits in PD patients still remains poorly understood. Huisman et al. (2004) showed a significant increase of dopamine expression in the olfactory bulb of hyposmic PD patients. It means that the dopamine in hyposmic PD brains does not necessarily decrease. Single-photon emission computerized tomography (SPECT) studies have reported that olfactory deficit is highly associated with the nigrostriatal dopamine transporter (DAT) binding abnormalities (Siderowf et al., 2005). DAT decline is closely associated with the worsening motor severity (Bohnen et al., 2016). Neuroimaging studies suggested that putamen associated with the bradykinesia-rigidity subscore is strongly affected in PD patients with olfactory dysfunction (Politis et al., 2018; Roos et al., 2019). These findings are in line with our observation. The greater decrease in nigrostriatal DAT binding in hyposmic PD patients indicates that these patients are likely to have a weaker response to dopaminergic medications and need a higher dose of LEDs. PIGD phenotype has been previously shown to be associated with more diffuse neurodegeneration (Fereshtehnejad and Postuma, 2017). Our study demonstrated that hyposmic PD patients were more likely to be classified as PIGD than normosmic patients.

Our study demonstrated that both normosmic and hyposmic PD patients had significantly reduced the NMSS scores at the follow-up period, and we suspect a dominant role of levodopa medications here. There was no significant difference in NMSS score between normosmic PD patients and hyposmic ones. RBD is likely associated with neurodegeneration in the pontine and medullary regions (Valencia et al., 2018). Hyposmia and RBD do not share the same neuroanatomical substrates. Although we observe trends toward a higher prevalence of RBD in hyposmic PD patients, these did not reach statistical significance. This result is in line with that of Hong et al. (2015). Emotional dysfunctions, such as depression and apathy, are very common in PD. Although there are several common neuroanatomical substrates for olfactory and emotional information processing, such as the amygdala, hippocampus, insula, and orbitofrontal cortex (Soudry et al., 2011), the relationship between olfaction and emotional dysfunction in PD remains controversial (Morley and Duda, 2011). Several studies suggested that olfactory dysfunction correlated with apathy (Hong et al., 2015; Masala et al., 2018b). However, our study found no significant relationship between olfaction and depression, which is in line with the observation of Morley et al. (2011). Because depression often occurs in advanced PD, longer-duration prospective cohort studies will be needed to clarify the potential association between olfaction and depression in PD.

Recent studies revealed a relationship between olfactory dysfunction and decline in both global cognition and specific cognitive domains, including episodic verbal learning, verbal memory, executive function, and attention (Baba et al., 2012; Fullard et al., 2016). However, our baseline data is inconsistent with those of previous studies. This discrepancy may be explained by the different age range of the patients. Our study demonstrated that hyposmic patients had worse cognition at year 2, and olfactory dysfunction somewhat promoted the development of cognitive impairment during the course of the disease. Hyposmic PD patients showed a faster deterioration in global cognition, especially in attention and calculation domain, as measured by MMSE. Considering the effect size of olfactory dysfunction was small, a longer-duration prospective study is warranted to investigate the impact of hyposmia on cognitive function. Cecchini et al. (2019) indicated that olfactory function was worse in PD patients with visuospatial dysfunction, but we did not find any similar relationship in our study. It may be because PD patients in our study were in an earlier stage, in which visuospatial skills were preserved. Greater deterioration in cognition was associated with worse odor identification and general olfactory function. It has been suggested that attention impairment was more closely correlated with PD dementia than memory loss (Baba et al., 2012). Bohnen et al. found a positive correlation between odor identification performance and forebrain cholinergic pathway integrity in PD patients. Progressive cholinergic denervation seems to play an important role in the development of cognitive decline, particularly in the domains of attention and execution (Bohnen et al., 2010; Fullard et al., 2016). Our study demonstrated that the longitudinal deterioration in cognition was associated with the poor odor identification. This result is in keeping with that of a previous study that odor identification seems to be more associated to cognitive central pathways connecting to the orbitofrontal cortex, piriform cortex, and amygdala, while odor threshold is related to individual differences of the nasal cavity (Masala et al., 2019). While the value of olfactory dysfunction as a biomarker for other NMSs is unclear.

## Conclusion

Taken together, olfactory dysfunction is one of the earliest NMSs of PD, and it can be used as a potential marker of disease progression. Our prospective study demonstrated that hyposmic PD patients showed a relatively worse course of PD progression compared with normosmic PD patients. Olfactory dysfunction was associated with worse motor symptoms and higher LED needs. Additionally, olfactory dysfunction may be a useful predictor for future cognitive impairment.

## Data Availability Statement

The raw data supporting the conclusions of this article will be made available by the authors, without undue reservation.

## Ethics Statement

The studies involving human participants were reviewed and approved by Medical Ethics Committee of Xiangya Hospital. The patients/participants provided their written informed consent to participate in this study. Written informed consent was obtained from the individual(s) for the publication of any potentially identifiable images or data included in this article.

## Author Contributions

RH, YZa, YH, YZo, JY, XiZ, LZ, XuZ, ZL, QX, QS, XY, BT, and JG contributed to the material preparation and data collection. RH, QS, JT, XY, BT, and JG performed the data collection analyses. RH, QS, and JG written the first draft of the manuscript, and all authors commented on previous versions of the manuscript. All authors contributed to the study conception and design. All authors read and approved the final manuscript.

## Conflict of Interest

The authors declare that the research was conducted in the absence of any commercial or financial relationships that could be construed as a potential conflict of interest.

## Footnotes

1 http://pd-mdcnc.com:3111/
